# Patient involvement in quality improvement – a ‘tug of war’ or a dialogue in a learning process to improve healthcare?

**DOI:** 10.1186/s12913-020-05970-4

**Published:** 2020-12-02

**Authors:** Carolina Bergerum, Agneta Kullén Engström, Johan Thor, Maria Wolmesjö

**Affiliations:** 1 Faculty of Caring Science, Work Life and Social Welfare, University of Borås, S-501 90 Borås, Sweden; 2 Jönköping Academy for Improvement of Health and Welfare, School of Health and Welfare, Jönköping University, P.O. Box 1026, S-55111 Jönköping, Sweden

**Keywords:** Clinical microsystem, Co-production, Healthcare organisation, Patient involvement, Quality improvement

## Abstract

**Background:**

Co-production and co-design approaches to quality improvement (QI) efforts are gaining momentum in healthcare. Yet, these approaches can be challenging, not least when it comes to patient involvement. The aim of this study was to examine what might influence QI efforts in which patients are involved, as experienced by the patients and the healthcare professionals involved.

**Methods:**

This study involved a qualitative design inspired by the constructivist grounded theory. In one mid-sized Swedish hospital’s patient process organisation, data was collected from six QI teams that involved patients in their QI efforts, addressing care paths for patients with transient, chronic and/or multiple parallel diagnoses. Field notes were collected from participant observations during 53 QI team meetings in three of the six patient processes. Individual, semi-structured interviews were conducted with 12 patients and 12 healthcare professionals in all the six QI teams.

**Results:**

Patients were involved in QI efforts in different ways. In three of the QI teams, patient representatives attended team meetings regularly. One team consulted patient representatives on a single occasion, one team collected patient preferences structurally from individual interviews with patients, and one team combined interviews and a workshop with patients. The patients’ and healthcare professionals’ expressions of what might influence the QI efforts involving patients were similar in several ways. QI team members emphasized the importance of organisational structure and culture. Furthermore, they expressed a desire for ongoing interaction between patients and healthcare professionals in healthcare QI.

**Conclusions:**

QI team members recognised continuous dialogue and collective thinking by the sharing of experiences and preferences between patients and healthcare professionals as essential for achieving better matches between healthcare resources and patient needs in their QI efforts. Significant structural and cultural aspects of performing QI in complex hospital organisations were considered to be obstructions to progress. Therefore, to sustain learning and behaviour change through QI efforts at the team level, a deeper understanding of how structural and cultural aspects of QI promote or prevent success appears essential.

**Supplementary Information:**

The online version contains supplementary material available at 10.1186/s12913-020-05970-4.

## Background

Patients and healthcare professionals carry valuable experiences of the healthcare system, which can contribute to quality improvement (QI), safer healthcare and further research [1–7]. Thus, service co-production and co-design are increasingly important healthcare development, and healthcare organisations are expected to involve relevant microsystems in QI interventions [8–13]. For healthcare QI, patients may be involved at different levels: individual, group, organisational and societal [5, 6, 10]. In Sweden, The Patient Act [14] regulates and strengthens patients’ positions and nudges healthcare organisations to work more actively with new methods to increase quality and safety at the different levels. However, the term ‘patient involvement’ lacks one definitive definition and, instead, involves many aspects and issues that are not clearly understood by patients, healthcare professionals, managers or other stakeholders [12]. It is difficult to define and measure quality because there is no shared understanding for what it is among patients and healthcare professionals [15]. Furthermore, patient involvement does not always provide what it appears to promise and does not automatically lead to better quality or safer care. In the healthcare context, professionals may behave paternalistically, leaving patients disempowered [3]. Such encounters may lead to value destruction rather than value creation [16].

Improvement science concerns how to conduct QI to narrow the gap between current practice and the best possible practice. It focuses on ‘what works’ to improve quality as well as the best ways to capture and spread lessons learned to encourage positive change. Its systematic and continuous actions, which lead to measurable improvements, fit well with the improvement efforts of complex healthcare systems. Therefore, it may inform the design, and re-design, of healthcare services [17–21].

This study rests on the premise that the healthcare system consists of clinical microsystems (CMSs) [22]. Formed around common purposes or needs, CMSs are the smallest, functional units in which patients and healthcare professionals meet and exchange information – for example in a delivery room, a primary care centre or in QI efforts. The patients and healthcare professionals involved are interdependent, share information and work together to co-produce quality, safety and cost outcomes at the frontlines of healthcare. CMSs are nested in meso- and overarching macro-systems, making macro-system outcomes depend on CMS outcomes. Therefore, to improve and sustain quality in a healthcare system, key leverage points exist at the CMS level [22]. In this study, the ‘CMS’ encompasses patients, next of kin, patient representatives and healthcare professionals, who are jointly involved in healthcare QI efforts in a hospital organisation.

QI in healthcare can be challenging when involving patients at the various healthcare levels [5, 10]. As mentioned above, the term ‘patient involvement’ includes many aspects and issues and is not clearly understood [12]. Additionally, there is a relative lack of empirical evidence about how it might work in QI interventions [10]. Patient involvement may also be complex and challenging due to, for example, patient frailty or other conditions limiting patients’ ability to participate [23]. Therefore, QI facilitation must be flexible and sensitive to each intervention’s context, both individually and at the group level [10, 24, 25]. The aim of this study is, therefore, to examine what might influence inter-departmental hospital process QI teams when involving patients in QI efforts, as experienced by the QI team members.

## Methods

This study has a qualitative design inspired by the constructivist grounded theory approach [26–28] which evolved from the original Glaser and Strauss grounded theory research methodology [29]. The authors were interested in ‘what is happening’ [26, 30], and the comparative and interactive nature of the constructivist grounded theory approach allowed for simultaneous collection and analysis of data to answer emerging empirical questions. Thereby, the subsequent gathering of data was informed by the analysis of previously gathered data. Furthermore, the constructivist grounded theory [26], allowed the researchers to relate to their contextual understanding while attempting to withhold theoretical preconceptions.

This study is based on field notes from 53 QI team meetings and on 24 semi-structured, individual interviews with participating patients, next of kin, patient representatives and healthcare professionals. This research is part of a larger project studying patient involvement in the QI of hospital organisations from CMS [22] and leadership [15, 31] perspectives.

### Setting

The context of this study is a patient process organisation in a mid-size, regional hospital in southern Sweden. This hospital provides healthcare in all specialities. The patient process organisation is a model applied for patients with common, serious illnesses, who are dependent on the inter-professional cooperation across clinics in the hospital and between hospital and related community-based services. For example, there are patient processes defined for patients admitted to the hospital with suspected hip fracture, sepsis or stroke, in which inter-professional cooperation across clinics are identified to be crucial for the each patient’s outcome. A patient process is often identified from the hospital’s patient safety work. It is formalised in an application procedure, and mandated by the hospital board. The aim for each defined patient process is to improve the patient care process. In this context, QI efforts are carried out at the group and organisational levels (i.e., in the healthcare mesosystem) [5, 10, 22]. Each defined patient process consists of an inter-professional QI team led by a physician (i.e. the process leader). During this research period, the hospital organisation under study consisted of 12 defined patient processes. Patients were involved in QI teams of six of the 12 patient processes. In some of the QI teams, they actively participated as representatives, while, in other teams, they were consulted with or interviewed by healthcare professionals but did not regularly attend the team meetings.

### Participants

To identify interview informants, the present authors employed purposive sampling, which allowed the sampling strategies to be flexible throughout the research process [26]. As mentioned above, six out of the 12 patient processes actively included patients in their QI efforts. These, therefore, were selected for the study. One of the authors (CB) attended 53 team meetings with three of the six selected QI teams. Table 1 offers a description of the QI teams (Table 1). In the following, the term ‘CMS’ encompasses these QI teams, consisting of patients, next of kin, patient representatives and healthcare professionals that are jointly involved in healthcare QI. Individual patients, next of kin, and patient representatives (designated, in the following, as ‘patients’), participated actively in eight of the team meetings CB attended. The total number of participants in the team meetings varied from two to 11. Healthcare professionals attending the team meetings included physicians; nurses; psychologists; counsellors; occupational, physical and speech therapists; pedagogues of special educational needs (designated, in the following, as ‘healthcare professionals’); students; development leaders; and team secretaries. Team meetings occurred once or twice a month for each QI team. The field notes were gathered between March 2016 and March 2019.

**Table 1:** Description of the QI teams included in the study and the data collection

	Field studies	Interviews
QI teams (CMS)	Neuropsychiatric diagnoses team Multi-diagnosed older persons team Pneumonia team ∑ = 53 team meetings	Breast cancer team Diabetes team Neuropsychiatric diagnoses team Multi-diagnosed older persons team Pneumonia team Prostate cancer team ∑ = 24 interviews

Patients and healthcare professionals from all six selected QI teams were asked to participate in individual interviews. These semi-structured interviews were conducted with 12 patients (7 females and 5 males) and 12 healthcare professionals (10 females and 2 males). At the time for the interview, patients were 25–81 years old, and the healthcare professionals were 37–77 years old. Interviews were performed between March 2017 and February 2019.

### Data collection methods

Field notes during team meetings were spontaneous and free, written with a focus on patient involvement in social interactions, communication, considerations, and decision procedures, whether or not the patient was physically present. CB had the opportunity to interact with the QI team members in a joint learning process during the QI efforts and, in this way, was able to observe the different contexts of meanings, actions, routines and practices involved. The field notes complemented the interviews and were also used during data analysis.

The individual interviews were conducted in locations chosen by the informants. For patients, the interviews were performed at the hospital, at the university, or in their homes. The average duration of each interview was 48 min. For healthcare professionals, the interviews were conducted at their work places. One hour was scheduled for each interview, and the average time used was 41 min. The interview guides (Additional file 1, Additional file 2) were semi-structured, with suggested, open-ended questions aiming at uncovering experiences, perspectives, motives and attitudes regarding patient involvement in healthcare QI [32]. For this study in particular, the focus was on how approaches to QI involving patients were integrated into the QI teams, and how this integration was experienced by the team members involved. All interviews were audio-recorded and transcribed verbatim. Data collection was terminated when two of the patient processes under study concluded their work. At that time, no new topics could emerge in the field notes or interviews, and the theoretical saturation was, therefore, considered to have been reached.

### Data analysis

The data analysis was performed in Swedish, and translated into English when writing the report. The quotes were translated and back-translated by English and Swedish natives, respectively. In line with the constructivist grounded theory approach [26], the analysis began with the first field notes from the QI team meetings, interview planning, and the selection of the first informants. Emerging empirical questions informed the content of subsequent interviews.

**Fig. 1:**
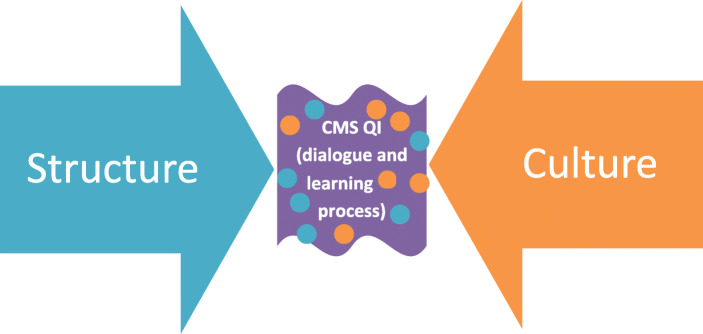
A model for illustrating how structure and culture in this hospital organisation were experienced by the QI team members in their QI efforts

The interviews were transcribed and read by two of the authors (CB and AKE) to gain a sense of the whole. Initial codes were coded by hand into an Excel spread-sheet and were then discussed among the authors. The codes were condensed in the same Excel spread-sheet following focused and theoretical coding. Memo-writing played a central role throughout the analytical progress [26, 27]. Categories were articulated inductively (Table 2) and confirmed using the grounded theory approach of constant backtrack comparison. These categories were then transformed into three concepts, leading to a model (Fig. 1) for understanding what might influence QI efforts involving patients in a hospital patient process organisation, as experienced by QI team members.

**Table 2:** Examples of data analysis in the study

Data	Initial coding	Focused coding	Theoretical coding	Category	Concept
*“If it’s considered to be so important, perhaps they should create the conditions that it takes, it actually takes a great deal of time... a great deal of time… if they think it is that important, yes.”* (Healthcare professional 1).	The management demands, but provides no support	A request for organisational support	Organisational support	Organisational support	Organisational structure – complexity
*“...it’s clear that... doctors and all those, they perhaps think in one way and patients think in another, and it is clear that it… it… they get my perspective on the whole thing, if you know what I mean…”* (Patient 4).	Healthcare professionals think in one way and patients think in another way	Patients and healthcare professionals think in different ways	Patients observe things that healthcare professionals don’t	Patients observing things that healthcare professionals don’t	Organisational culture – learning capability
*“But that, that implies that one can have... a dialogue with, with the staff, and that they are accepting of that. But ultimately, it’s up to them to decide, you know. But that one can… inject that which one has experienced personally… both experienced and learnt, and… well.”* (Patient 13).	The dialogue is important for patient involvement	Patient involvement presupposes a dialogue	Interaction between patients and healthcare professionals	Interaction between patients and healthcare professionals	Interaction – dialogue

## Results

The six studied QI teams took different approaches to involving patients in their QI efforts. In three teams, patients actively participated as team members, and in the remaining three, they were consulted, or interviewed, by healthcare team members but did not regularly attend the team meetings (Table 3). The QI team members’ expressions of what might influence QI involving patients concerned the organisational structure, culture, and the desired interaction between patients and healthcare professionals in healthcare QI efforts (Fig. 1).

**Table 3:** Description of patient involvement approaches in the QI teams

Patient involvement approach	Teams
Patient representatives participating regularly	Breast cancer team Diabetes team Neuropsychiatric diagnoses team
Patient representatives invited on one occasion	Prostate cancer team
Individual interviews with patients in a structured way	Pneumonia team
Individual interviews and workshop with patients	Multi-diagnosed older persons team

### Organisational structure

Although the hospital patient process organisation under study was designed to bridge well-known complexity challenges and streamline the interconnected patient care processes, the QI team members repeatedly referred to practical obstacles in their QI work. Much of this was due to the complexity and hierarchy of the organisational structure. Overall, both patients and healthcare professionals experienced a lack of clarity and transparency within the organisation. They said organisational changes were frequent, with some team members expressing uncertainty about how long their own QI team would actually last. For example, some informants described how their patient process and, subsequently, the QI team, was terminated without a formal explanation that all team members could understand. Another QI team made an unexpected break due to the process leader’s resignation. In both cases, some of the QI team members felt their QI work was cancelled just as they were starting to get somewhere. In other patient processes, team meetings were often cancelled at the last minute, in some cases leaving patients uninformed about what was happening. High staff turnover—especially in the QI teams, but also, generally, in the hospital—impaired the teamwork overall by creating a sense of uncertainty and hindering the work’s sustainability. The QI team members expressed that they could understand the organisational problems to some extent, but, nevertheless, it did create frustration and uncertainty within the teams. Healthcare professionals described a work situation in which they were too busy to properly engage in QI and were forced to prioritise clinical work over QI projects. Similarly, patients recognised and reflected upon the workloads of the healthcare professionals.

Patient (P11): *No … if one compares with sport and so forth, with ice hockey, it’s not possible to play flat out for the whole match, it doesn’t work. It has to be a good pace, so to speak. People have to recover, and come back, and be able to relax and so forth.*

Despite increasing expectations from the hospital management for QI teams to involve patients in their work, the informants considered management support to be poor. The QI team members recognised the complexity of involving patients in healthcare QI and how their QI efforts might benefit from various and adjusted types of facilitation, yet their impression was that the hospital management only provided directions for carrying out QI and expected quick results. This was especially highlighted by those QI team members who felt their QI work had been abruptly cancelled. Hence, there was a general hunger for organisational guidance and facilitation.

Healthcare professional (V12): *It* [QI efforts involving patients] *does take time, it needs some space to settle. And people keep working, and one doesn’t have the proper preconditions. You just work like you always have.*

Hierarchy played another important role for the QI team members but was expressed in different ways. Patients, who were consulted or were interviewed by teams on single occasions, were mainly concerned about the way hierarchy and inequality might affect patient–healthcare professional care meetings and relationships at the individual level. They said this might, in turn, affect the individual’s ability to be involved. Furthermore, they reasoned that the severity of this barrier could be correlated to patients’ ages and personal characteristics. For example, hierarchy was argued to be more of a barrier to involvement for older people. Hierarchy was also expressed as being correlated to the attitudes of the healthcare professionals, which was exemplified in how routines could be adapted and managed, from an operational rather than patient-oriented stance, around patients.

In the QI teams where patients were regularly involved, patients and healthcare professionals were more equally concerned about how hierarchy might affect patient involvement in their teamwork. Nevertheless, experiences differed greatly. Some patients had higher expectations of having influence over the QI work than did others, expressing everything between satisfaction and frustration with their role as representatives on the team. A patient, who had several years of experience as a regular team member, expressed this barrier of hierarchy:

Patient (P5): *There is a hierarchy in the healthcare system, I’ve gradually come to understand that doctors still have that attitude. I mean “they are doctors, I’m the patient”, “the patient doesn’t know what’s in their best interests”. The managers want to explain, because they are the ones in charge. Yes, they are supposed to see the big picture, but we don’t have a hope of influencing them.*

Healthcare professionals were generally more satisfied with the degree of patient involvement in the QI efforts than patients, and described the team relationships as more equal, though some of them desired increased equality.

Patient involvement in healthcare QI was also discussed as a potential threat for individual patients, if not managed carefully. The QI team members recognised that patients, depending on diagnoses, could find themselves placed in both an influential and a vulnerable position at the same time. Multiple examples of engagement involving stressful situations for patients were pointed out. For example, healthcare professionals stated that patients with neuropsychiatric diagnoses could easily engage themselves in things that would, simultaneously, be stressful for them.

Healthcare professional (V6): *It’s a bit odd* [for the patient] *to simultaneously have a position and a vulnerability.*

However, conflictingly, both patients and healthcare professionals were adamant that they were the ones responsible for handling such situations.

### Organisational culture

According to the experiences of the QI team members in this study, patient involvement in QI efforts was also influenced by the attitudes, maturity levels, learning capabilities and levels of responsibility taking within the organisational culture.

Both patients and healthcare professionals reflected upon the ongoing mind-set shift towards person-centred care in healthcare. Some believed it was a cultural shift that had already begun, while others requested that it happen. Nevertheless, there was a general agreement that in order to gain sustainable outcomes, a holistic approach to patient perspectives, rather than professional or operational perspectives, should guide healthcare QI.

Patient (P14): *They can’t just see my part of the puzzle, they have to see the whole picture regarding people who are old and sick.*

Some healthcare professionals described feelings of guilt and shame in situations during QI efforts when failings in the organisation were revealed. However, they did not recognise that patients were often already aware of these organisational failings. The more regularly involved the patients were in the QI teams, the higher was their awareness. However, patients never took an accusatory stance. Rather, they expressed willingness to contribute to and improve the organisation, claiming that their involvement in QI was even more important. In line with patients’ views, none of the healthcare professionals was satisfied with the current organisational strategy, which, they believed, was mainly focusing on operational efficiency and cost reductions. They would prefer prioritising their time on patient involvement and value creation.

Healthcare professional (V14): *I think, a lot of the time, here in the hospital, it’s “yes, now he/she has been booked in for this and that examination, so let’s do that treatment later, and then we will do like this, and then we will do like that, and so on“. But has someone asked? Has anyone explained the alternatives* [to the patient]*?*

Interviews and field notes also revealed an opposite side to this agreement on patient involvement and value creation. On the one hand, patients involved as team members were satisfied with the QI teams’ work, but, on the other hand, they struggled with understanding the organisation, grasping the purpose of the QI efforts, and identifying their roles in the teams. Comparatively, healthcare professionals were generally more satisfied with the way patient preferences were represented in their teams, regardless of what approach to patient involvement was applied. Some healthcare professionals reasoned they were doing as they were told and appeared most interested in ticking the box of patient involvement. Others struggled and put effort in how to relate to, organise for, proceed with and manage patient involvement.

Some of the QI teams were more experienced with involving patients than others, and some patients were more experienced with being involved in healthcare QI than others. Therefore, the expectations of how QI actually worked, or should work, differed greatly. Generally, the degree to which the patients were involved met their own expectations. For example, patients who were consulted on one occasion did not have high expectations for the outcomes of the subsequent QI efforts, while patients who were highly engaged desired a higher degree of influence, co-production and feedback within the QI team. The latter group of patients highlighted the concern that patient representation might become merely symbolic.

Patient (P5): *Sure, I’m involved, and they listen, and I say my piece, I’m allowed to comment on their. But, when afterwards something should come of it* – *do my comments count for anything, or do they just do what they had planned all along?*

QI team members had different views about who was responsible for healthcare QI. While some argued that QI work was a responsibility solely for healthcare professionals, others stressed the need for shared responsibility in QI efforts at all levels of the healthcare organisation. The risk of patient needs exceeding healthcare resources was a frequent argument in favour of healthcare QI being co-produced.

Healthcare professional (V7): *If the demands are too great, those patients have to help so that we can meet those demands. In that case it isn’t just our responsibility, because if we are working together it becomes a little fifty-fifty.*

QI team members described an attitude within the healthcare organisation of trivialising patients’ expressed needs and positioning patients in dependency. This was seen as an obstacle to patient involvement in QI. Additionally, the way healthcare professionals considered themselves ‘experts of the field’ and believed their perspectives could, therefore, take precedence over those of patients, was considered a barrier by patients and healthcare professionals. Both patients and healthcare professionals problematized patients’ experiential knowledge but did not necessarily agree on its importance in healthcare QI. For patients involved in QI teams concerning chronic diagnoses, experiential knowledge was a strongly emphasized contributor to QI. Healthcare professionals also agreed with this. Some even described patients’ experiential knowledge, and the work of identifying patient needs, as setting the directions for efficient QI efforts in the patient process and as being part of clinical and professional development.

Healthcare professional (V3): *A lot that is important for many working in, involved with healthcare, that isn’t always what is important for the patient.*

However, most interviewed healthcare professionals still considered their expert knowledge to be superior.

Furthermore, both patients and healthcare professionals problematized the complexity of harmonising healthcare resources to patient needs on every level of the healthcare organisation. The general agreement, in the interviews and in field notes, was that increased patient involvement in healthcare QI, by identifying ways to better match healthcare resources to patient needs and make improvements toward that goal, would be cost-effective and lead to better healthcare outcomes in the long-term.

Patient (P6): *I don’t think it would put greater demands on the healthcare system, I really don’t think that. I think, on the contrary, that it might actually become easier for the healthcare professionals to also know “what, what does this patient want?” Instead of guessing.*

### Interaction

The QI team members reasoned that patient involvement in QI efforts was an ongoing process of interaction. In the patient process organisation, shared knowledge, roles and relationships, and dialogue were considered to be prerequisites for this desired interaction.

Patients regularly involved in the QI teams talked about the efforts they put into understanding the professional language and how the whole organisational system, including QI, functions. These efforts were time consuming and demanding, yet the patients considered them to be educative and vital to ensure patient influence in the QI efforts. During the learning process, patients acknowledged the relationship between their own knowledge about healthcare and the professional expertise. This further encouraged the patients to bring forward their experiences and opinions. A next-of-kin explained how this learning process contributed to the ability to point at mismatches and QI areas:

Patient (P6): *I have realised how, how they think in healthcare, in a way. And I have been able to say, to explain how I, as a relative, think. It doesn’t always match up.*

However, the healthcare professionals did not recognise this process of knowledge acquisition that patients were gaining possession of, and the importance the patients put to it. Although stating they were sincerely interested in learning from patients, and wanted to step out of their professional role in the process, most healthcare professionals were mainly interested in the collection of patient preferences and priorities only, to apply it in QI initiatives by themselves. Actively involving patients throughout the QI work process was not usually considered an option.

Healthcare professional (V4): *When I go to that kind of meeting, I am not there in my professional capacity. Rather, I attend to try to capture an experience and understand “what are we missing?*

The future of patient involvement in QI efforts was reflected upon in a lively manner. QI team members’ reflections mainly concerned shortages they had experienced within healthcare—situations in which patients could have contributed even more. There was a general agreement that healthcare QI could consist of a collaboration of patients and healthcare professionals, equally participating in mutual engagement. Patients who had experience of being regularly involved in such QI efforts in other contexts described their participation as an ongoing process, developing from representing fellow patients via cooperating with the healthcare organisation to co-producing with the healthcare organisation. This process was believed to fill a relation gap between the healthcare organisation and patients.

Patient (P10): *That’s when we move from* [patient] *members’ care, towards co-operation, towards working together* [co-production]*. We take an active role and fulfill a need.*

Some healthcare professionals did note the existence of this gap but focused more on operational efforts to harmonise healthcare resources with patient needs. Yet, some did believe the solution was to be found in a new relationship with patients.

Healthcare professional (V9): *They say that the patient is the focus, but the patient should be a participant in the process.*

Across the interviews, QI team members often reflected upon how to start a form of dialogue between patients and healthcare professionals at different levels of healthcare organisations, and particularly in QI efforts. As mentioned above, healthcare professionals were mainly interested in operational efforts to harmonise resources to meet patients’ needs, and identifying instruments to measure outcomes of such QI efforts. To them, the ideal situation would have involved QI team members being able to identify areas of improvement and address them, deficiency by deficiency, so that they would not appear again.

Healthcare professional (V10): *But with a dialogue, I think that one could avoid a great deal of misunderstanding, among other things, and perhaps arrive at something more participatory, somewhere halfway.*

Patients reasoned more about how effective healthcare QI efforts with better outcomes depends on their involvement in an interactive dialogue process.

Patient (P13): *But that, that implies that one can have a dialogue with the staff, and that they are accepting of that. But ultimately, it’s up to them to decide. But that one can inject that which one has experienced personally* – *both experienced and learnt.*

A desired interaction and dialogue between patients and healthcare professionals was argued as being essential for the learning process in healthcare QI efforts, and this idea concluded most of the interviews. One of the healthcare professionals in the study summarized the importance of patient involvement:

Healthcare professional (V14): *It* [patient involvement] *preserves his/her* [the patient’s] *intrinsic value*.

## Discussion

This study found that QI team members’ expressions of what might influence their QI efforts involving patients mostly concerned organisational structure and culture. QI team members also desired interaction and dialogue in a learning process between patients and healthcare professionals in healthcare QI efforts.

### Performing QI in complex healthcare organisations

As outlined in the methods section, the hospital patient process organisation is designed to manage complexity-related challenges and promote QI efforts in interconnected patient care processes. However, the QI team members in this study experienced their organisational context as being unpredictable and inexplicit when providing guidance for patient involvement, which created constant uncertainty that affected the QI process. They stressed that cancelled QI team meetings, constant staff turnover, and, in a couple of cases, the termination of patient processes, weakened the system in which they were assigned to perform QI, thereby affecting their performance and experience. This has also been argued in similar organisational contexts [33]. To overcome and compensate for this, some patients worked out useful strategies, such as making the effort to learn the professional language used in the QI teams and trying to understand how the healthcare system works. The extant literature has proposed that healthcare organisations can be considered complex adaptive systems [34, 35]. As such, they can, in a positive manner, be described as dynamic, continually responding and adapting to internal and external influences. However, they can also demonstrate slowness and inertia, with embedded behaviours remaining unchanged and even large-scale attempts to transform the organisation failing to re-design the system or dislodge undesired existing norms [36]. This study has added knowledge of how the complexity of healthcare organisations may affect the way QI efforts work (or fail to work), as experienced by patients and healthcare professionals at the CMS level.

Considering patient preferences in QI efforts, which, for some QI teams in this study, was a new and uncharted approach, added further challenges to this already complex system. First, the approach included many novel aspects and issues, and no clear definition for the term ‘patient involvement’ was provided to which the QI team members could relate [12]. Secondly, there was a relative lack of guiding empirical evidence and organisational support [2, 10], leaving QI team members struggling with overarching questions about how to organise for patient involvement in their QI efforts. Thus, the QI team members were occupied with considering how to balance roles and relationships, how to achieve mutual learning, how to measure the impact of patient involvement and how to match the knowledge of patient needs to the resources within the patient processes. At the same time, the QI team members considered organisational support to be absent, saying that management did not facilitate the ongoing learning process they were experiencing. Earlier research has pointed out that participants who experience the greatest success of QI efforts are those who actively work on them, but peripherally-involved managers and healthcare professionals believe the same QI efforts have limited impact [31]. Perhaps there was a similar lack of dialogue between organisational levels in the current setting, leading to poor understanding of what was actually happening in the QI teams, and affecting management decisions to terminate patient processes.

Despite a relative lack of empirical evidence, a few examples of successful patient involvement in QI efforts were published in the literature at the time of the study. These examples concerned clarity about delineating the rationale for QI efforts, identifying the right model for achieving specific outcomes, defining clear roles and responsibilities for the QI team members, facilitating partnership, ensuring meaningful involvement, and supporting the behavioural changes that follow [1, 10, 24]. However, in the present case, existing research evidence was neither known nor applied by patients, healthcare professionals or the management in the patient process organisation.

### Dialogue, collective thinking and organisational learning

Despite insufficient knowledge of how patients could best contribute, QI team members realised the potential patients had for adding a significant dimension to complete the perspectives important for QI [10, 24]. The more involved patients and healthcare professionals became with each other during QI, the more they mutually understood the capacity of co-production [10, 31]. However, patients and healthcare professionals did have different views of how to actually co-produce. While the importance of combining patients’ experiential knowledge with professionals’ expert knowledge [37] was enthusiastically reflected upon by patients, healthcare professionals did not fully recognise these as two equally important knowledge bases. In this matter, healthcare professionals’ opinions were somewhat ambiguous. Some emphasized the contribution of patients’ experiential knowledge, but did not show any interest in transforming it into co-productive actions. Others preferred taking an empirical stance, questioning the representativeness of single patients [38] in QI teams and claiming to possess the prior right to the expertise. Thus, patients’ complementary knowledge risked being left out [39]. This inequality may be one of the basic barriers to commence interaction and dialogue within the QI teams, and, subsequently, a barrier to creating patient-centred value from QI efforts. This might also be one of the reasons patients expressed fears about their representation becoming tokenistic, which, in turn, may affect their motivation to be involved. This issue has previously been noted as a major challenge for healthcare QI efforts [39, 40]. Considering both patients’ and healthcare professionals’ views in this study, it was discernible that a ‘tug of war’ existed over whose knowledge was to take precedence.

Furthermore, QI team members in this study identified a gap between the outcomes of healthcare and patient needs. Patients viewed this gap to be more severe than healthcare professionals. However, both patients and healthcare professionals urged for large-scale organisational change from an operational–professional led to a more patient–preference led management. This mismatch between the needs of the organisation and the needs of the patients has been previously addressed [38] as creating a dissonance between objective processes and subjective experiences. In the present study, the main conclusions were that healthcare should be organised around patients, rather than around diagnoses and operations. Furthermore, QI team members agreed that, to be sustainable, QI efforts should emanate from patient needs in a learning system. The current linear thinking they were experiencing in the patient process organisation, with the application of top-down tools, such as issuing more policies and regulations, was argued against. Instead, QI team members requested a strategy to support a learning system [41] containing feedback loops to gently move towards behaviour change. However, as previously stated [39], there was disagreement over who possessed the correct knowledge about what patients actually needed. Some healthcare professionals expressed anxiety that increased patient involvement would lead to patients presenting unrealistic wish lists for QI.

Another risk, proposed by healthcare professionals, was that patient involvement in QI could put frail patients in even more vulnerable positions. Whether these arguments were expressions of them being considerate of, or threatened by, patients getting ‘too’ involved, can be interpreted as another ‘tug of war’, but needs to be further investigated. However, according to patients, this risk was nothing to be concerned about. They expressed awareness of their current roles in the QI teams in relation to their frailty or diagnoses, of the limited available resources in the organisation, and that areas for improvement could be redirected to other organisational levels [5, 6]. Instead, they expressed increased interest in providing, based on individual ability, ideas for improvement to create a greater value of healthcare for themselves and other patients.

**Fig. 2:**
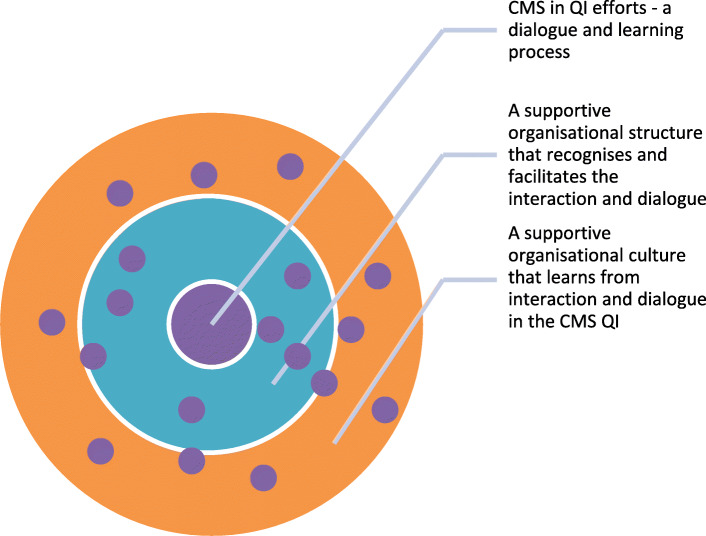
A model for illustrating a new desired mode in which learnings from the dialogue in the QI teams function as an integral part of a supportive structural and cultural context of hospital organisations

In summary, QI team members desired the creation of a new relationship between patients and healthcare professionals. This has been previously discussed in the literature [1, 10, 13]. In this study, it was suggested mainly at the individual level in QI teams, but also at other levels of the organisation [5, 42, 43]. Yet, such a relationship presupposes justice and equality both within QI teams, and between QI teams and their supporting organisational structure. Results of this study point at several structural and cultural barriers to interaction and dialogue that are experienced by QI teams. Hierarchy, knowledge and evidence recognition, organisational transparency, and management guidance are aspects of justice and equality that need to be addressed and put into place to promote the desired process [10, 11, 25, 44–46]. Furthermore, this study suggests that the more patients were regularly involved face-to-face in QI teams, the more developed were the attitudes, maturity thinking, learning capabilities, and levels of responsibility taking by both patients and healthcare professionals. In the QI teams with patients regularly involved, team members reasoned that, to develop organisational learning resulting in better quality and safer care, joint efforts from patients and healthcare professionals are essential. And for this to happen, QI teams requested fitted facilitation to establish the partnership and collective thinking that would promote the necessary dialogue [10, 11, 25, 44–46] for determining patient-oriented forms of care and quality measurements in their patient processes, in QI efforts and in outcomes [11, 46]. The desired dialogue was expressed as an ongoing process, moving from a state of consultation, via involvement, to partnership and shared leadership in co-producing healthcare. This reasoning compares well with earlier reasoning in the extant literature [43], in which levels of patient involvement is put on a continuum moving from consultation, involvement, to partnership and shared leadership. Subsequently, this study suggests this continuum to be of relevance also for QI efforts. The more involved with each other patients and healthcare professionals get, the more the partnership and shared leadership in co-production is valued and desired. Thus, Fig. 1 displays how organisational structure and culture in the hospital patient process organisation under study is experienced by the QI team members, while Fig. 2 illustrates a desired mode. In this model, the organisational structure and culture embraces, facilitates and supports learnings from QI in which patients are involved, contributing to a learning organisation.

In line with other research [15, 47, 48], the results of this study suggest the importance of further organisational research about how to manage patient involvement in QI efforts to generate evidence-based guidance, and on how to apply this guidance to support healthcare QI teams when involving patients. For patients and healthcare organisations, the present authors believe this involvement to be a fundamental basis for development.

### Strengths and limitations

One strength of this study was the data collection method, in which the field notes, based on participant observations, complemented the individual interviews. Another strength was the data analysis method, with interviews being transcribed by hand in Word documents and analysed, for transparency, using an Excel spread-sheet throughout the process. The patients and healthcare professionals interviewed were another strength, as they represented diverse medical diagnoses, ages, professions, and different forms of patient involvement applied in the work of the QI teams in the patient process organisation. Another strength, especially for data analysis, was the professional background of the research team, with competences in medicine, nursing, social work/social care, grounded theory, participatory research methods, patient/user involvement and healthcare QI efforts.

However, a limitation of this study is the small number of QI teams that involved patient preferences in their QI work and, therefore, were included for data collection. This led to a limited number of patients and healthcare professionals being asked to participate in the study. Nevertheless, the qualitative approach provided a suitable platform for informants to share useful information about their experiences, in line with study aims. Adding to this limitation, two of the included patient processes were terminated and concluded their work during the study period, which, in turn, terminated the data collection and may have affected the study’s ability to reach theoretical saturation. However, the authors did include all QI teams that had experience with involving patients in their QI efforts in the hospital patient process organisation under study, and the QI team members were asked to participate in an interview, even after the termination of their patient process.

The researchers were interested in learning from this particular patient process organisation to improve future QI efforts in the same or other contexts, making transferability of the results one of the study’s purposes. One author was equipped with much pre-understanding (i.e., background knowledge about the services and context under study) and held a position at the hospital, but not in the patient process organisation under study. This may have affected the dependability of the study and cannot be ignored. Still, some pre-understanding was necessary to gain access to and understand the QI team meetings, to interact with the team members, and to conduct the study. Therefore, and due to the scarcity of new information in the field notes and the interviews, the authors considered the field notes and interviews sufficient for examining what might influence QI efforts involving patients in a hospital patient process organisation, as experienced by the QI team members.

To strengthen the study’s validity, quotations from the interviews were highlighted. Furthermore, the discussions within the QI teams that contributed to the field notes and offered valuable information concerning topics to cover in the interviews, also provided useful input to the data analysis and additional validation to the study’s findings. Also, the continuous backtracking of emerging concepts during the analysis, according to constructivist grounded theory [26, 27], strengthened the validity of the results.

During the translation process, the quotations highlighted in this report were translated and back-translated by English and Swedish natives, respectively. Altogether, the authors believe these actions strengthened the findings’ trustworthiness.

## Conclusions

This study found that the QI team members recognised continuous dialogue and collective thinking by the sharing of experiences and preferences between patients and healthcare professionals as essential for achieving better matches between healthcare resources and patient needs in their QI efforts. Significant structural and cultural aspects of performing QI efforts in complex hospital organisations were considered to be obstructions to progress. Therefore, to sustain learning and behaviour change through QI efforts at the CMS level, a deeper understanding of how structural and cultural aspects of QI promote or prevent success appears essential.

## Supplementary Information

**Additional file 1.** Interview guide. Patients, next-of-kin, patient representatives. English version.

**Additional file 2.** Interview guide. Healthcare professionals. English version.

## Data Availability

The dataset analysed for this article is available only to the researchers involved in the project. This is due to privacy protection issues and in accordance with Swedish and European legislation, and the Regional Ethical Review Board in Gothenburg, Sweden. The Excel spread-sheet used in the analysis is available from the corresponding author on reasonable request.

## Abbreviations

CMS: The clinical microsystem
QI: Quality Improvement
