# Supplementary material for: Patient involvement in quality improvement – a ‘tug of war’ or a dialogue in a learning process to improve healthcare?
Source: BMC Health Serv Res. 2020 Dec 2;20:1115. doi: 10.1186/s12913-020-05970-4 (PMC7709309; doi:10.1186/s12913-020-05970-4)
Supplement: Supplementary file 1 — Additional file 1. Interview guide. Patients, next-of-kin, patient representatives. English version. [file 12913_2020_5970_MOESM1_ESM.docx]

#
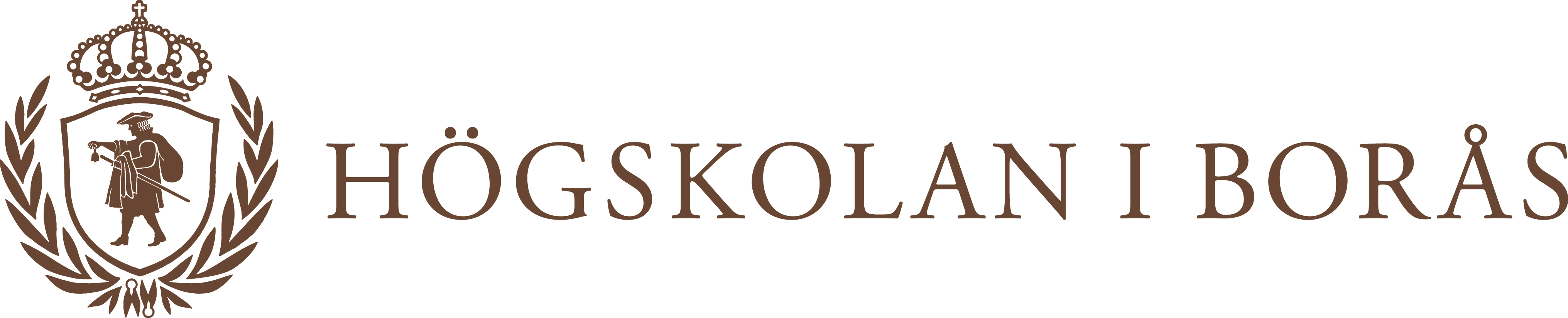

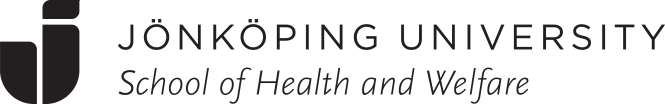


# Interview guide

# Patients, next-of-kin, patient representatives.

Information about the study and the research project.

Consent to participate.

**Initial short survey with background data**

- Gender
- Age
- Patient/next-of-kin
- For next-of-kin: relation to the patient

**Questions (individual interview)**

- Please tell me about your experiences of the quality improvement work that you have participated in. Please start from the beginning, when you were asked to participate.
- How would you describe your participation to someone who does not know?
- Please describe the most important parts of the quality improvement project and why they were important.
- Please tell me what you believe your participation has contributed to in the quality improvement project.
- What are your previous experiences of patient involvement in healthcare? If you have previous experiences, what are the similarities and differences?
- What difficulties are there to participating in quality improvement projects? Do you have any ideas for improvement?
- What are your positive experiences of participating?
- Tell me what you think will happen in the quality improvement project. What will it lead to?
- Please describe what you think may hinder or enable it (what you have described) to happen.
- What does patient involvement in healthcare mean to you (definition)? Please give examples.
- Do you know what the different national policy documents and guidelines on patient involvement say? How do you understand them?

**Supplementary questions when necessary**

- Can you please give me an example?
- What do you mean by that?
- Please develop your thoughts on...
- Please tell me more of...
- Can you please explain?
- Have I understood it right that…?

**Concluding questions**

- Is there anything else you want to add before we finish the interview?
- Can I contact you again if necessary?

Thank you for participating!
